# Progression of the COVID-19 pandemic, Brazilian healthcare workers’ emotional burden and the effects on professional fulfillment at the end of the third wave: a longitudinal study

**DOI:** 10.3389/fpsyt.2023.1237123

**Published:** 2023-11-03

**Authors:** Sonia Regina Loureiro, Antônio Waldo Zuardi, Isabella Lara Machado Silveira, José Alexandre de Souza Crippa, Jaime Eduardo Cecílio Hallak, Flávia de Lima Osório

**Affiliations:** ^1^Ribeirão Preto Medical SchoolUniversity of São Paulo, Ribeirão Preto, Brazil; ^2^National Institute for Science and Technology (INCT-TM, CNPq), Brasília, Brazil

**Keywords:** mental health, health occupations, longitudinal study, COVID-19 pandemic, burnout

## Abstract

**Introduction:**

Even though the long-term effects of the COVID-19 pandemic on healthcare workers’ mental health remain unknown, such effects might negatively impact health services and patient safety, especially in countries like Brazil, where there is little investment in public health policies.

**Objectives:**

To assess how the mental health indicators of Brazilian healthcare workers progressed between the beginning and 2 years after the pandemic (at the end of the third wave when there was a significant decrease in the number of new cases and deaths).

**Methods:**

The sample comprised healthcare workers whose mental health indicators have been monitored since the beginning of the pandemic in Brazil. The potential participants were addressed via social media and contacted through class councils and health institutions across Brazil. A total of 165 participants answered instruments at the baseline and 2 years after the pandemic. Data were collected online using the Redcap platform and addressed symptoms of anxiety, depression, post-traumatic stress, insomnia, and burnout (emotional exhaustion, depersonalization, and professional fulfillment).

**Results:**

Brazilian healthcare workers faced three periods of intensified incidence of new cases and deaths due to COVID-19 for 2 years. Approximately one-third of the sample still experiences high levels of anxiety, depression, and post-traumatic stress. Insomnia indicators remained the most prevalent compared to the baseline assessment, while post-traumatic stress symptoms (*p* = 0.04) and professional fulfillment (*p* = 0.005) decreased.

**Conclusion:**

The lack of positive changes in mental health indicators coupled with decreased professional fulfillment over time highlights the pandemic’s chronic effects and the need for organizations to monitor these workers’ mental health, especially in developing countries like Brazil, where there is a high demand for health services and public policies are poorly structured and unstable.

## Introduction

Even though the long-term effects of the COVID-19 pandemic on healthcare workers’ mental health remain unknown, such effects may negatively impact health services and patient safety. The COVID-19 pandemic was considered an extreme event, characterized by chronic stress due to how it progressed. Meanwhile, most studies portrayed the pandemic’s most immediate and acute effects on the mental health of the general population and healthcare workers (1), while just a few implemented medium/long-term follow-ups to address the emotional conditions of healthcare workers and monitor how indicators and associated factors progressed. Hence, most studies were restricted to a cross-sectional assessment at one point in time during the pandemic (2). Additionally, Fattori et al. (3) note a lack of longitudinal studies addressing healthcare workers’ mental health, especially during the second year of the pandemic, which prevents comparisons between the problem indicators at the pandemic’s beginning and at the end of the third wave, when it came into control.

Therefore, this study is intended to fill in this gap. It is part of a more extensive study called MENTALvid addressing Brazilian healthcare workers to identify how mental health indicators concerning anxiety, depression, post-traumatic stress, insomnia, and burnout have progressed during the pandemic, considering different epidemiological contexts.

Two waves of COVID-19, involving different epidemiological periods, were compared in this study. The results show that the stress resulting from the high number of cases and deaths influenced the mental health conditions of Brazilian health professionals (4). The same is reported by Lamb et al. (5), who assessed a cohort with 22,501 English healthcare workers between April 2020 and August 2021. They note that mental health symptoms varied over the 17-month follow-up, with a higher prevalence when the health systems were under more significant pressure because of increasingly higher monthly mortality rates caused by COVID-19.

In addition to different epidemiological periods, various other conditions have been associated with more significant or lower emotional burden among healthcare workers. These include the reorganization of health services’ resources to meet new needs (6), the availability of vaccines (7), and the need to care for patients with other pathologies, whose demands were suppressed during the pandemic’s critical phases (8). Moreover, work overload, associated with burnout, has been associated with a desire to quit the job (9), sick leaves, and early retirement, in addition to workers moving to less risky careers offering more benefits (10).

Note that contextual economic and financial factors emerged as overload factors for the population in general and healthcare workers as the pandemic persisted over time. Worsened economic and financial conditions led individuals to experience insecurity, negative professional prospects, and financial concerns due to changes in income and daily routine, configuring as some of the pandemic’s collateral effects (11).

Healthcare workers have long been vulnerable to mental health problems (12). Hence, the emergence of the COVID-19 pandemic had an even more intense impact on these professionals, whether because of increased workload and overload, the need to stay distant from their families, greater risk of contamination, loss of patients, or even changes in working dynamics, and workers having increased contact with unfamiliar situations and considerable uncertainty. Such a context favored more emotional problems among healthcare professionals, who stood out among the most vulnerable to mental health problems (13).

Studies conducted around the world reported high rates of anxiety, depression, insomnia, post-traumatic stress, and burnout indicators among healthcare professionals, revealing extreme emotional distress and vulnerability (14). Based on the analysis of the 44 meta-analyses addressing mental health indicators presented by hospital teams during the COVID-19, Dragioti et al. (15) report a general prevalence rate of anxiety symptoms of 29.90, 28.44% of depression symptoms, 39.45% of insomnia or sleep disorders, 44.30% of stress, and 18.75% of post-traumatic stress. Ghahramani et al. (16) performed a meta-analysis of 30 papers on the burnout prevalence among health professionals working during the COVID-19 pandemic. They identified a general rate of 52%, with 51% of exhaustion indicators, 52% of depersonalization, and 28% of decreased personal/professional fulfillment.

Considering such a context and acknowledging that the pandemic’s harmful impacts on the mental health of healthcare workers have consequences over the long term, negatively affecting health services and patient safety (17), we deem it relevant to assess mental health indicators over the long term, addressing different epidemiological contexts and points in time, especially after the end of the pandemic’s critical period. As it remained a global public health emergency, standing guidelines were recommended for long-term pandemic management (18).

This study’s objective was to evaluate how indicators of anxiety, depression, post-traumatic stress, insomnia, and burnout progressed 2 years after the pandemic, characterized by the end of the third wave when new cases and mortality rates decreased significantly. The baseline, the first wave of the COVID-19 pandemic, was analyzed to verify the impact of emotional burden on healthcare workers’ professional fulfillment. The specificities of the epidemiological context of the pandemic in Brazil justify this study (19). For example, according to the Brazilian Ministry of Health (20), the pandemic is characterized by a high number of cases (more than 30 million) and high mortality rates (660,000 deaths) in addition to a high rate of deaths caused by COVID-19 among healthcare workers, mainly physicians and nursing workers.

The following question guided this study: Will Brazilian health workers present fewer mental distress and burnout indicators 2 years after the pandemic when there is a favorable context characterized by a decreased number of deaths from COVID-19? The hypothesis was that Brazilian health professionals would experience lower rates of mental distress and increased professional fulfillment following the favorable progression of the pandemic.

## Methods

### Sampling and sample size

Convenience sampling was adopted. Hence, the participants were recruited through social media, TV, radio, and from class councils and important health institutions in different Brazilian regions. The study’s objectives, invitation letter, and a link to access the platform and data collection instruments were provided to the target population, and those interested would access the link and be directed to the Redcap platform. Vanderbilt University developed Redcap to collect, manage, and disseminate research data; it also allows the development of online databases (21). The participants would first access free and informed consent forms and then complete self-report instruments. Inclusion criteria were: (a) being a Brazilian health worker, providing care to patients with COVID-19 (self-report), and (b) digitally signing the free informed consent form to confirm voluntary participation. This study included all the participants who met these criteria; the sample size was not previously defined, given that it was not possible to estimate, *a priori*, the number of health professionals who worked to care for patients with COVID-19, at the beginning of the pandemic in advanced.

The sample comprised Brazilian frontline workers. A total of 916 participants were included in the baseline [please see Osório et al. (22) and Supplementary material for further information on sampling]. All the participants who completed the instruments at the baseline were recruited for data collection at D720. Of these, 770 participants did not complete the instruments at D720 and were excluded from the analysis. Among the 165 who completed all the instruments in the first part of data collection, four stopped working in the health field, and 15 did not complete all the instruments in the second part and were excluded. Therefore, 146 participants remained in this study.

### Instruments

The following instruments were used in data collection:

- Generalized Anxiety Disorder-7 (GAD-7): a 7-item self-report instrument that screens anxiety-associated symptoms. It was proposed by Spitzer et al. (23) and validated in Brazil by Moreno et al. (24) (*α* = 0.92; sensitivity/specificity = 0.89/0.82 for cut-off ≥10). The instrument’s reliability for the sample addressed here was *α* = 0.91.
- Patient Health Questionnaire-9 (PHQ-9): a 9-item self-report instrument to assess depression indicators. It was proposed by Kroenke et al. (25) and validated in Brazil by Osório et al. (26) (sensitivity/specificity = 1.00/0.98 for cut-off ≥10). The reliability for this sample was *α* = 0.90.
- Posttraumatic Stress Disorder Checklist for DSM-5 (PCL-5): self-report instrument used to assess symptoms of posttraumatic stress disorder using the criteria established by the DSM-5 (27). Its short version (8 items), which was translated, adapted, and psychometrically assessed by Osório et al. (28) and Pereira-Lima et al. (29), was used (*α* = 0.93; ICC = 0.84; sensitivity/specificity = 0.97/0.61 for cut-off ≥14). The instrument’s reliability for the sample addressed here was *α* = 0.92.
- Insomnia Severity Index (ISI): 7-item self-report instrument intended to assess the severity of insomnia in the last 2 weeks (30). It was adapted and validated in Brazil by Castro (31) (*α* = 0.87; sensitivity/specificity = 0.73/0.80 for cut-off ≥8), with a reliability equal to 0.89 (alpha de Cronbach) for the current sample.
- Abbreviated Maslach Burnout Inventory–Human Services Survey (aMBI-HSS): to assess burnout syndrome (dimensions of emotional exhaustion, depersonalization, and personal fulfillment). Its abbreviated version (22 items), proposed and validated among health professionals (32, 33), was used. Cut-off scores ≥9 indicate emotional exhaustion, ≥ 6 depersonalization, and ≥ 10 indicate professional accomplishment. 22 (*α* = 0.65–0.94). A reliability (alpha de Cronbach) between 0.82 and 0.88 was found.

### Data collection

The data collected for this specific study occurred at two different points in time, which portrayed different epidemiological contexts of the pandemic in Brazil. The first data collection (baseline) occurred between May and September 2020. According to the Ministry of Health, it was characterized by the critical phase of the pandemic’s first wave, with 3.6 deaths per 10,000 inhabitants. The second data collection (D720) occurred 2 years later, between May and September 2022, a time characterized as the end of the pandemic’s third wave, when there were 0.1 deaths per 10,000 inhabitants.

Therefore, the participants were required to have completed the instruments at the baseline to be included in the D720 phase. All the participants included in the baseline received a personalized link to access the data collection protocol concerning the D720 phase via WhatsApp or e-mail, according to the participant’s preference.

### Data analysis

The cutoff points proposed by Brazilian psychometric studies were adopted to identify emotional burden/emotional problem indicators (GAD-7): ≥ 10 (24); PHQ-9: ≥10 (26); PCL-5: ≥14 (29), ISI: ≥8 (31); aMBI-HSS: ≥9 emotional exhaustion, ≥6 depersonalization, and ≥ 10 professional fulfillment (33). Besides the participants’ risk perceptions, information on sociodemographic and occupational data was also collected.

Data were stored in the Redcap platform and statistically analyzed using Statistical Package for the Social Sciences (SPSS Statistics 20). The participants’ sociodemographic, occupational, and clinical information (i.e., gender, age, psychiatric treatment, occupation, public/private hospital, COVID-19 frontline, concern with being infected, satisfaction with protective measures) concerning the baseline and 720 days after were compared using Chi-square (for nominal data) or Student’s *t*-test (for interval data). We considered the cutoff points of each instrument for analysis of the outcome indicators. Participants who scored higher than the recommended for each self-rating scale were considered to present indicators of the specific outcome. We used the Wilcoxon signed-rank test (a non-parametric test for paired nominal data) to compare the number of participants with indicators in each evaluation phase. The percentage of participants was used in the figure to clarify the results. The statistical significance was set at *p* ≤ 0.05 for all the analyses.

### Ethical considerations

This study is part of the MENTALvid study, initiated in May 2020. It was submitted to and approved by the Institutional Review Board at the Hospital das Clínicas, Medical School, University of São Paulo at Ribeirão Preto—USP (CAAE: 30691020.8.0000.5440; Process 4.187.877). The participants received informed consent forms and signed them digitally.

## Results

A sample of 146 participants was effectively included in this study. Table 1 shows the sociodemographic characteristics of the participants included (*n* = 146) and not included (*n* = 770) in the D720 phase. The participants’ profile was compared to verify potential selection bias.

**Table 1:** Characterization of the participants in phase D720 who were included (*N* = 146) and those who were not included (*N* = 770).

Variables	% of respondents who completed the instruments on day 720		Statistical test value	*P*
	Yes (*N* = 146)	No (*N* = 770)		
	N (%)	N (%)		
Gender			χ^2^ = 2.224	0.136
Female	123 (84.2)	607 (78.8)		
Male	23 (15.8)	163 (21.2)		
Age—mean (SD)	39.78 (9.6)	37.1 (34.1)	*F* = 0.072	0.789
Lives alone			χ^2^ = 0.037	0.848
Yes	25 (17.4)	129 (16.7)		
No	121 (82.6)	641 (83.3)		
Psychiatric treatment			χ^2^ = 1.305	0.253
Yes	19 (13.0)	76 (9.9)		
No	127 (87.0)	694 (90.1)		
Occupation			χ^2^ = 2.685	0.101
Nurse	51 (34.9)	325 (42.2)		
Other	95 (65.1)	445 (57.8)		
Works in a public hospital			χ^2^ = 0.291	0.590
Yes	111 (76.0)	601 (78.1)		
No	35 (24.0)	169 (21.9)		
Works in the COVID-19 frontline			χ^2^ = 0.299	0.585
Yes	110 (75.3)	598 (77.7)		
No	36 (24.7)	172 (22.3)		
Is concerned with being infected				
Yes	116 (79.5)	612 (79.5)	χ^2^ = 0,000	0.994
No	30 (20.5)	158 (20.5)		
Is satisfied with protective measures			χ^2^ = 0.399	0.527
Yes	120 (82.2)	649 (84.3)		
No	26 (17.8)	121 (15.7)		

Statistical significance at *p* ≤ 0.05.

The sample addressed in the D720 phase was also predominantly composed of women, aged 39 on average, who lived with a partner; 13% of the participants reported having received psychiatric treatment before the onset of the pandemic. Approximately 35% of the sample was from the nursing field, and the remaining participants were physicians, psychologists, physical therapists, nutritionists, occupational therapists, or dentists. Most worked in the public sector as frontline staff and were concerned about virus infection. Approximately 80% reported concerns about being infected with the virus, and 17.8% were not satisfied with the protective measures provided by the employing institution.

In the sample described above, the same characteristics predominated (for example, a higher percentage of women from the nursing area), according to an analysis of the profiles of the people who were excluded from the D720 phase. As a result, even though there was a significant sample loss, the results in Table 1 show that there was no selection bias because there was no statistically significant difference between the participants in this phase and those who withdrew from the study (*p* > 0.101).

**Figure 1:**
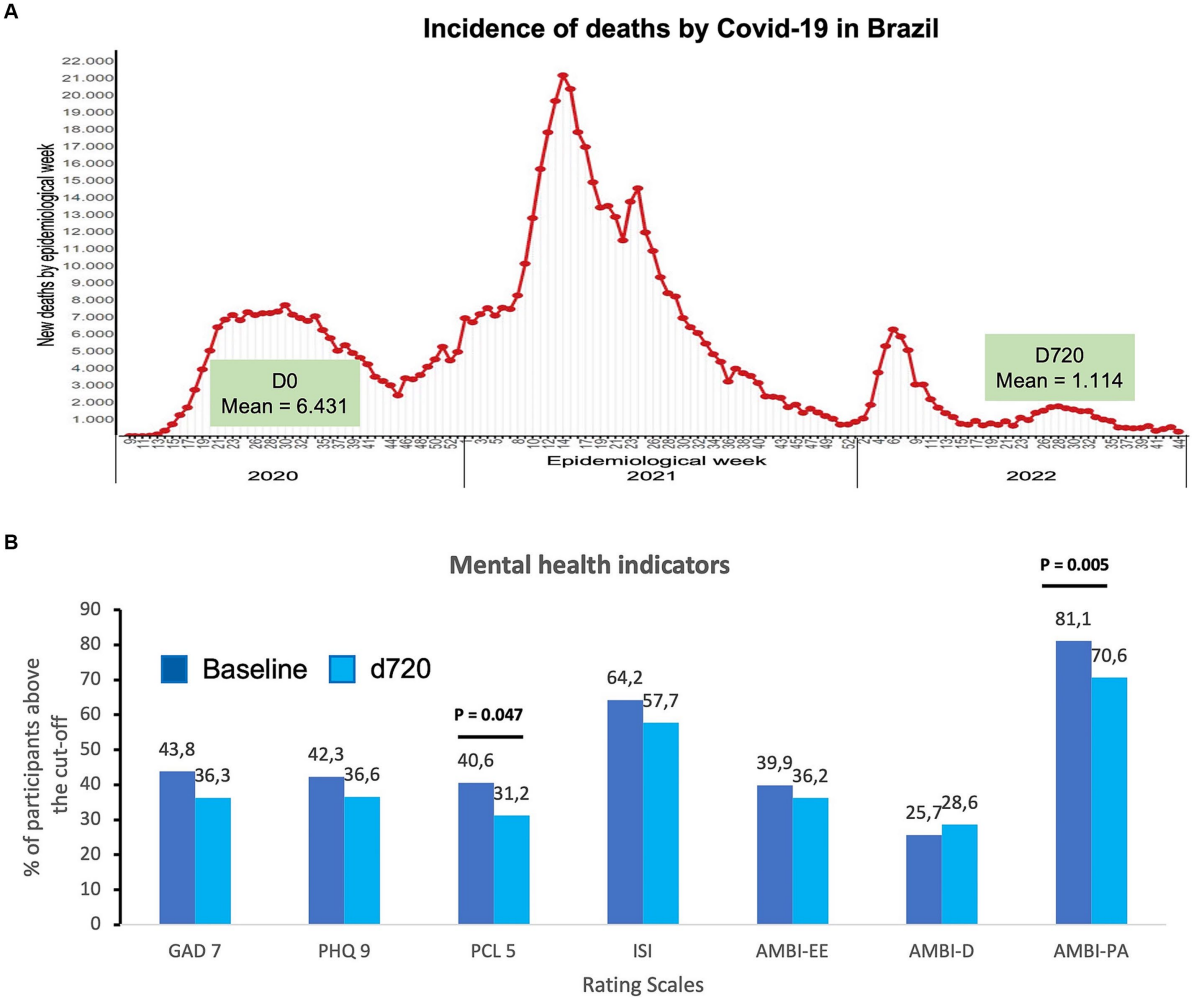
**(A)** Distribution of the deaths caused by COVID-19 in Brazil from 2020 to 2023, highlighting the average number of deaths in the two data collections (D0 and D720). (adapted from Brazilian Ministry of Health (https://infoms.saude.gov.br/extensions/covid-19_html/covid-19_html.html). **(B)** Percentage of participants with scores above the cut-off in self-rating scales for anxiety (GAD-7), depression (PHQ-9), posttraumatic stress (PCL-5), insomnia (ISI), and three dimensions of burnout, emotional exhaustion (AMBI-EE), depersonalization (AMBI-D), and personal achievement (AMBI-PA). The value of p was established at *p* < 0.05 to determine statistically significant differences using the Wilcoxon signed-rank test.

In this two-year follow-up, health teams faced three periods in which the incidence of new cases and deaths from COVID-19 intensified. Figure 1A shows the death rates from COVID-19 in the Brazilian population between 2020 and 2022, with peaks in epidemiological weeks 31st of 2020, 14th of 2021, and 6th of 2023. Data collection from the baseline phase (May to September 2020, corresponding to epidemiological weeks 20th–40th) coincided with the first wave of deaths (average of 6,431 deaths per week), while data collection from the D720 phase, which occurred between May and September 2023 (epidemiological weeks 20th–40th), was marked by a significant decrease in deaths (average 1,330 deaths/week), with statistical significance (*F* = 43,315; *p* < 0.001).

Regarding mental health indicators, Figure 1B presents the percentage of participants who scored above the cutoff points in the self-report instruments according to the data collection phases. The results show that the mental distress indicators were numerically lower than in the D720 phase (a time characterized by greater control over the COVID-19 pandemic). However, they are not statistically different from the baseline (first wave) and remained high. Approximately one-third of the sample still experience high levels of anxiety (36.3%), depression (36.6%), and post-traumatic symptoms (31.2%). At the baseline (first data collection), the percentages were 43.8, 42.3, and 40.6, respectively). Only the decrease in post-traumatic stress was statistically significant (PCL-5: 40.6% first wave vs. 31.2% end of the third wave; *p* = 0.047). Insomnia indicators remained the most prevalent, with more than half of participants showing signs, which remained statistically stable (64.2% in the first wave and 57.7% at the end of the third wave). Regarding burnout signs, emotional exhaustion and depersonalization rates remained stable (39.9% vs. 36.2% for exhaustion, 25.7% vs. 28.6% for depersonalization). Additionally, professional fulfillment dropped significantly (*p* = 0.005), with approximately 30% of the participants reporting dissatisfaction at this level (the percentage at the baseline was 18.9).

## Discussion

An analysis of the mental health indicators among Brazilian healthcare professionals 2 years after the COVID-19 pandemic revealed that the anxiety, depression, insomnia, burnout, exhaustion, and depersonalization rates identified in the first pandemic wave in Brazil remained high. Thus, the hypothesis that these indicators would decrease after the pandemic’s positive progression was not confirmed. Additionally, the indexes currently identified remained high and are higher than those reported by Chutiyami et al. (14). This finding suggests that these professionals still endure considerable distress and vulnerability despite the favorable changes in the epidemiological context.

On the other hand, these results differ from those reported by Fattori et al. (3), in which mental health indicators (general health, anxiety, and stress) significantly improved among Italian healthcare workers 24 months after the beginning of the COVID-19 pandemic. Additionally, the rates found in the present study (at the two different points in time) were considerably higher than those found by an extensive meta-analysis performed by Dragioti et al. (15), reporting 40% of sleep disorders, 30% of anxiety and depression, and 20% of post-traumatic stress.

Two indicators presented statistically significant changes 2 years after the pandemic: a lower rate of post-traumatic stress was found, but burnout remained higher, especially regarding professional fulfillment. The lower percentage of participants with post-traumatic stress is likely related to the end of the third wave, with an expressive drop in the number of cases and deaths (20), and consequently, less pressure imposed on healthcare services, greater mastery of supportive technical procedures, and more favorable clinical outcomes. Moreover, the availability of vaccines (7) and greater knowledge about the disease probably favored decreased post-traumatic stress rates, leading to desensitization regarding risks.

Still, as previously noted, the rates found in the study for post-traumatic stress were much higher than that reported by Dragioti et al. (15). Note that these results differ from Damico et al. (34), who conducted a multicenter study in Italy with ICU nurses. They found an increase in the prevalence of PTSD cases 12 months after the baseline data collection, without significant changes in anxiety or depression rates. In addition to the different epidemiological contexts verified as the pandemic progressed (11), these comparisons and divergences highlight the potential impact of macro conditions contributing to PTSD indicators.

Regarding burnout indicators, the emotional exhaustion and depersonalization dimensions did not change from the first assessment, while professional fulfillment indicators changed for the worse; i.e., they decreased significantly, suggesting higher job dissatisfaction. The burnout rates found in this study concerning exhaustion and depersonalization are lower than those reported by the meta-analysis performed by Ghahramani et al. (16), who analyzed studies conducted in 2020 at the beginning of the pandemic, which concerns this study’s baseline. Therefore, the fact that these rates remained high after 2 years of the pandemic, indicating a potential ceiling effect in the exhaustion and depersonalization dimensions, which did not change despite a decreased demand and differences in epidemiological contexts, suggests that other variables are at play. Perhaps these indicators remained high due to high rates of anxiety and depression, which is in agreement with the extensive review performed by Ulfa et al. (35). After analyzing studies conducted in 48 different countries, they concluded that the general burnout scores were associated with the presence of depression and anxiety.

Müller et al. (36) performed an international multicenter study. They verified increased emotional exhaustion and depersonalization between the first wave and the end of the second wave, suggesting that high levels of burnout accumulate over time. Similarly, Sexton et al. (37) addressed three pandemic waves in the USA and identified that emotional exhaustion increased since the beginning of the pandemic among the different groups of healthcare workers. They noted the potential impacts of this finding, considering that the healthcare workers’ increased emotional burden may have repercussions in the long run.

When specifically analyzing the effect of decreased professional fulfillment over time, we considered the observation of Zhou et al. (38) that the high work demands during the pandemic led to burnout, expressed by exhaustion and depersonalization, and decreased professional fulfillment. In addition, Ulfa et al. (35) find it extremely important to consider that decreased professional fulfillment is related to lower self-confidence, loss of enthusiasm, and lower productivity.

Zhou et al. (38) also note that the organizational support perceived by healthcare workers may play a protective role, favoring job satisfaction. This study did not directly assess organizational support in any of the points when data were collected; hence, this analysis is speculative but suggests that health organizations face difficulties in this sphere, failing to provide support at different levels. For example, a previous study (39) shows that 80% of Brazilian healthcare workers lacked institutional support.

The low levels of professional fulfillment found in this study are of concern, as workers tend to refrain from engaging with their work environment, possibly impacting the quality of care delivery (35). In this sense, high levels of depersonalization coupled with decreased professional fulfillment, characterized by a sense of incompetence and lower job satisfaction, affect the workers’ well-being and the future of healthcare delivery systems (40).

Hence, in agreement with Hill et al. (17), the care provided by healthcare workers is essential for the functioning and effectiveness of health services, and the long-term negative impact on professional fulfillment is likely to have more vast repercussions for society in general. Hence, this leads us to reflect upon the emergent need for organizational support to prevent more significant harm among professionals chronically exposed to occupational stress, considering the adverse working conditions existing before the pandemic, characteristic of developing countries like Brazil (41).

This study’s limitations concern: (a) an absence of sample calculation and the use of convenience sample, which may impact the results’ representativeness; (b) online data collection, which depends on the participants’ having access to computer/smartphone and internet; (c) relevant sample loss, even though the participants’ sociodemographic profile remained the same; (d) the use of self-report instruments, which enable the participants to report symptoms without diagnostic confirmation; (e) a lack of analyses considering the specificities of the participants’ organizational conditions; (f) the data analysis, which does not include associations between mental health indicators with other demographic and occupational variables; (g) a lack of previous assessment of the professionals’ mental health conditions, which would allow for more comprehensive comparisons; and the heterogeneity of professions from the health filed and a failure in reporting the medical specialties. This study’s strengths include comparing between the healthcare workers’ burden indicators in two contrasting epidemiological contexts: one measurement was taken 2 years after the initial critical phase of the pandemic in Brazil when there were high mortality rates in the general population and among healthcare workers.

The lack of positive changes in the mental health indicators and decreased professional fulfillment over time highlight the pandemic’s chronic effects. These findings imply the need for organizations to monitor these workers’ mental health, considering they continue to present high levels of distress with the potential to impact their professional practice. It is especially relevant among developing countries like Brazil, where there is a high demand for health services and public policies are poorly structured and unstable.

The data presented here may be relevant at the level of public policies, as they can inform the planning of prevention strategies in the face of future pandemics. Considering that different factors influenced the mental health indicators of health workers, the study data suggest the need for health institutions, at an organizational level, to balance workload and work shifts, as well as the flow of tasks, in a to reduce occupational overload and favor the provision of services safely for professionals and service users. The data also points to the need for care for this population, through institutional programs/ interventions that focus on identified points of mental health vulnerability, with support resources that can minimize damage, its evolution and future abandonment of the profession. Proposals for continuing education and institutional support, the latter involving access to psychological/psychiatric care for the most vulnerable and promotion of self-care behaviors in the workplace, may be relevant and guiding points for actions. Future research may be relevant to monitor this evolutionary process, highlighting the impact of adversities and the support offered, in order to elucidate and highlight their effectiveness.

## Data availability statement

The raw data supporting the conclusions of this article will be made available by the authors, without undue reservation.

## Ethics statement

The studies involving humans were approved by Comitê de Ética em Pesquisa em Seres Humanos do Hospital das Clínicas da Faculdade de Medicina de Ribeirão Preto - USP. The studies were conducted in accordance with the local legislation and institutional requirements. The participants provided their written informed consent to participate in this study.

## Author contributions

FO, AZ, JC, JH, and SL: conception and design and substantial contributions to drafting the article or revising it critically for important intellectual content, final approval of the version to be published. FO, IS, AZ, and SL: collect, analysis, and interpretation of data. All authors contributed to the article and approved the submitted version.
