# Supplementary figures and images for: Progression of the COVID-19 pandemic, Brazilian healthcare workers’ emotional burden and the effects on professional fulfillment at the end of the third wave: a longitudinal study
Source: Front Psychiatry. 2023 Nov 3;14:1237123. doi: 10.3389/fpsyt.2023.1237123 (PMC10657209; doi:10.3389/fpsyt.2023.1237123)

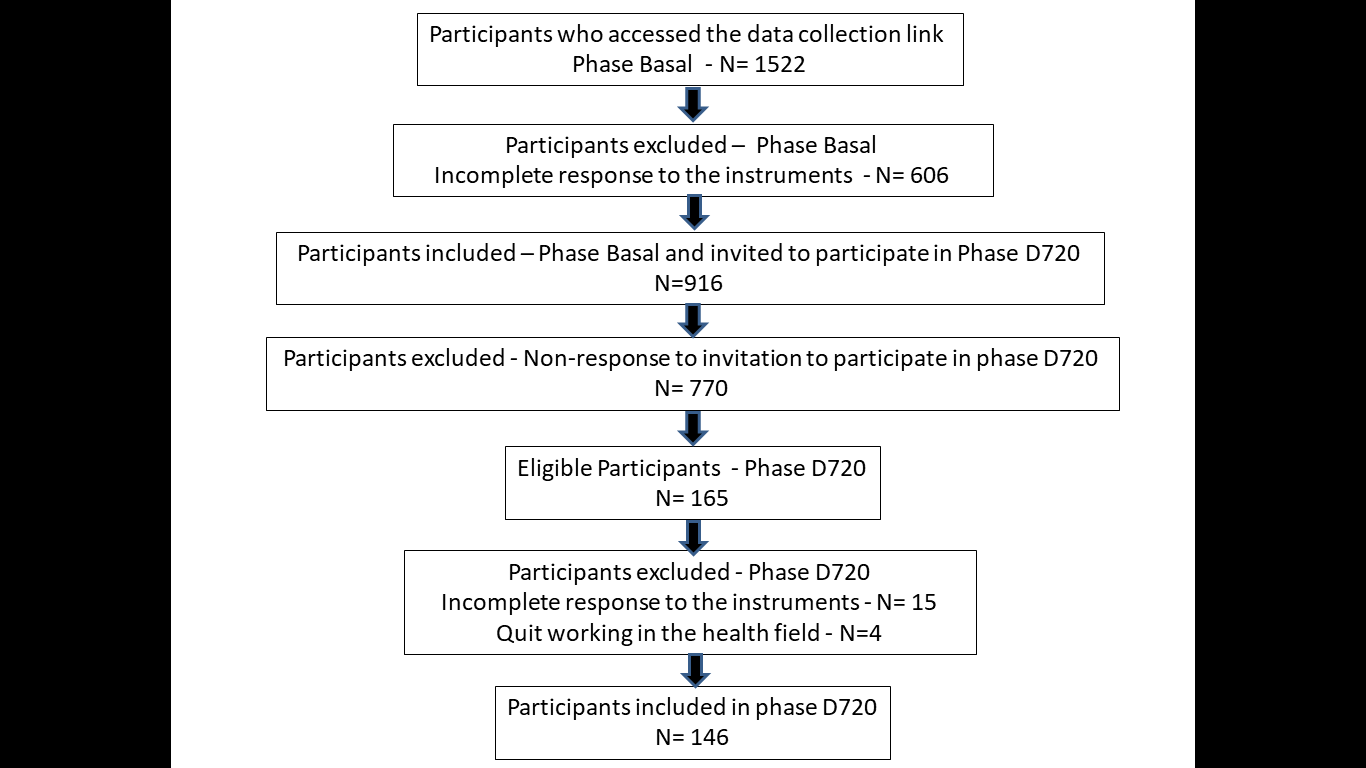


SM1 - Study sampling

Supplement: Supplementary file 1 [file Data_Sheet_1.docx]
